# Systemic Interleukin-4 Application Promotes Functional Recovery and Reprograms Neuroinflammatory and Molecular Responses after Spinal Cord Injury in Rats

**DOI:** 10.7150/thno.123815

**Published:** 2026-02-11

**Authors:** Obada Taleb Alhalabi, Stefan Heene, Guoli Zheng, Raban Heller, Tim Schubert, Marcin Luzarowski, Xiaowei Zha, Johannes Walter, Lea Hansen-Palmus, Bahram Biglari, Xing-Jin Wang, Laura Ruebenacker, Thomas Skutella, Karl Kiening, Christian Patrick Schaaf, Sandro Manuel Krieg, Andreas Wilhelm Unterberg, Klaus Zweckberger, Alexander Younsi

**Affiliations:** 1 Department of Neurosurgery, Heidelberg University Hospital, INF 400, 69120 Heidelberg, Germany.; 2 Medical Faculty, Heidelberg University, INF 672, 69120 Heidelberg, Germany.; 3 Institute for Experimental Endocrinology, Charité—Universitätsmedizin Berlin, corporate member of Freie Universität Berlin, Humboldt-Universität zu Berlin, Berlin Institute of Health, Anna-Louisa-Karsch-Straße 2, 10178 Berlin, Germany.; 4 Department of Food Chemistry and Toxicology, Technische Universität Berlin, Königin-Luise-Straße 22, 14195 Berlin, Germany.; 5 Department for Trauma Surgery and Orthopaedics, Reconstructive and Septic Surgery, Sportstraumatology, German Armed Forces Hospital Ulm, Oberer Eselsberg 40, 89081 Ulm, Germany.; 6 Institute of Human Genetics, Heidelberg University, INF 366, 69120 Heidelberg, Germany.; 7 Core Facility for Mass Spectrometry and Proteomics, Center for Molecular Biology at Heidelberg University (ZMBH), INF 329, 69120 Heidelberg, Germany.; 8 Institute of Virology, Technical University of Munich, Helmholtz Munich, Trogerstr. 30 81675 Munich, Germany.; 9 Department of Paraplegiology, BG Trauma Centre Ludwigshafen, Ludwig-Guttmann-Straße 13, 67071 Ludwigshafen, Germany.; 10 Department of Neuroanatomy, Institute for Anatomy and Cell Biology, University of Heidelberg, INF 307, Heidelberg, Germany.; 11 Department of Neurosurgery, Brunswick City Hospital, Brunswick, Salzdahlumer Straße 90, 38126 Braunschweig, Germany.

**Keywords:** Spinal cord injury, IL-4, neuroinflammation, functional recovery, cytokines, transcriptomics

## Abstract

**Rationale:**

Traumatic spinal cord injury (SCI) initiates a cascade of local and systemic inflammatory events that exacerbate tissue damage, hinder regeneration, and impair functional recovery. Interleukin-4 (IL-4) is an anti-inflammatory cytokine that promotes M2-macrophage polarization, but its functional benefit in SCI and the underlying mechanisms remain incompletely defined. We evaluated whether systemic IL-4 therapy can enhance recovery and modulate neuroinflammation in a rat model of SCI, and examined the translational relevance of key cytokine signatures in human SCI.

**Methods:**

Female Wistar rats (n = 120) were randomized to sham surgery, SCI with vehicle, or SCI with IL-4 treatment. SCI was induced at T10 by clip contusion-compression; IL-4 (0.5 µg/kg) or vehicle was administered intraperitoneally twice daily for up to 7 days post-injury (dpi). Functional recovery was assessed with the Basso-Beattie-Bresnahan (BBB) scale, CatWalk XT gait analysis, and gridwalk testing. Spinal cords collected at 1, 3, 7, 14, and 28 dpi underwent immunohistochemistry, RNA sequencing, and proteomic profiling. Serum cytokines were quantified in rats by bead-based multiplex assays and compared with longitudinal cytokine profiles from SCI patients.

**Results:**

IL-4-treated rats demonstrated significantly improved BBB scores and multiple CatWalk XT gait parameters by 14 dpi versus vehicle. RNA-seq and proteomics identified upregulation of pathways related to axonogenesis, tissue repair, and reduced TNF-α-mediated pro-inflammatory signaling. Immunohistochemistry confirmed increased IBA1⁺/ARG1⁺ and IBA1⁺/CD206⁺ M2-macrophages, reduced IBA1⁺/iNOS⁺ M1-macrophages, smaller cystic cavity area, and higher APC⁺ oligodendrocyte counts in IL-4-treated animals. Serum profiling showed suppression of acute/subacute pro-inflammatory cytokine surges (1-7 dpi) with IL-4. In SCI patients, lower circulating levels of these cytokines were associated with better neurological outcomes.

**Conclusions:**

Repeated systemic IL-4 administration after SCI promotes functional recovery, shifts macrophage polarization toward a regenerative phenotype, reduces astrogliosis and oligodendrocyte loss, and suppresses systemic inflammation. Multi-omics integration together with patient data suggests IL-4 targets convergent pathways of neuroprotection and immune modulation, supporting its further development as a therapeutic candidate for SCI.

## Introduction

Spinal cord injury (SCI) is associated with life-long disability and dependency, with incidence rates rising in recent years 1, 2. Advances in acute management have improved survival, but effective neuroprotective or regenerative treatments to mitigate long-term neurological deficits remain scarce. While the primary mechanical insult is irreversible in clinical practice, its extent critically influences recovery potential after SCI 3. In contrast, the secondary injury cascade—driven by biochemical and cellular processes unfolding over hours to weeks—offers therapeutic windows for intervention.

Local and systemic inflammatory responses are central to these secondary cascades. Pro-inflammatory cytokines rise sharply within hours after injury, peaking around 6-12 hours, and then gradually declining over several days after trauma 4. Subsequently, the activation of microglia, as well as the infiltration of neutrophils and peripherally derived macrophages amplify leukocyte extravasation, perpetuate inflammation, and drive progressive tissue destruction 5-9. The resulting neuroinflammatory milieu not only exacerbates functional decline but also impedes regeneration via glial scar formation 10. Consequently, modulating the post-SCI immune response toward an anti-inflammatory, pro-regenerative phenotype has been a major focus of experimental therapies 11, 12.

IL-4 is a potent anti-inflammatory cytokine that naturally shapes immune responses after traumatic brain injury (TBI) and SCI by promoting M2-type macrophage polarization 13-15. Exogenous IL-4 enhances the M2a-macrophage phenotype 16, which supports tissue repair via anti-inflammatory cytokine release, attenuates pro-inflammatory signaling, and facilitates axonal regrowth. Conversely, IL-4 deficiency worsens SCI outcomes 17. Prior studies administering IL-4 after SCI in rodents - whether systemically via intraperitoneal injection 18 or once intrathecally 48 h post-injury 19 - demonstrated modulation of neuroinflammation and neuroprotection, yet without conclusively linking IL-4 treatment to robust, sustained functional improvement.

Here, we present a comprehensive preclinical investigation of systemic IL-4 therapy in a refined rat model of SCI. By administering higher, repeated intraperitoneal doses than in earlier studies, we aimed to achieve greater CNS exposure and enhance therapeutic efficacy. We employed a multimodal battery of behavioral assays to detect subtle improvements in locomotion, coordination, and strength. Mechanistic insights were obtained through immunohistochemistry, transcriptomics, and proteomics of injured spinal cord tissue, while systemic immune modulation was profiled via high throughput seromics. Finally, to evaluate translational potential, we examined whether serum cytokine signatures relevant to IL-4's effects in rats were associated with functional recovery in SCI patients.

## Materials and Methods

### Study overview

We employed a thoracic clip contusion-compression injury model. A total of 120 adult female Wistar rats (250-300 g; Janvier Labs, France) were randomized to one of three groups: sham animals (laminectomy only; n = 30), SCI with IL-4 treatment (n = 45), or SCI with vehicle treatment (n = 45). Animals received intraperitoneal injections of recombinant rat IL-4 (PeproTech, 400-04) or sterile saline twice daily for up to 7 dpi (Supplementary Figures S1A and B). All behavioral, histological, and molecular analyses were conducted by investigators blinded to treatment group allocation.

Neurological recovery was evaluated with a battery of behavioral assessments capturing frequency, coordination, and strength of hindlimb movement prior to injury and at multiple intervals up to 28 dpi. Histological analyses were performed on spinal cord tissue collected after transcardial perfusion at 3 dpi and 28 dpi, representing subacute and chronic phases, to evaluate local immune responses using immunohistochemistry. To investigate systemic immune modulation, serum cytokine profiles were quantified at multiple timepoints using a high-throughput bead-based multiplex assay. Animals were housed under a 12-hour light/dark cycle at 26 °C with food and water available *ad libitum* and given an acclimatization period of 3-5 days prior to baseline behavioral assessments.

### Spinal cord injury and postoperative care

For all surgical procedures, anesthesia was induced and maintained with isoflurane (1.5-2.5%) in a 1:1 mixture of O₂ and N₂O. After microsurgical laminectomy over the T10 vertebra and randomization, a 28 g modified aneurysm clip (Fehlings Laboratory, Toronto, ON, Canada) was applied extradurally for 60 seconds to induce a contusion-compression SCI, as previously described 20-22. Rat belonging to the sham group underwent laminectomy only.

12 h post-operatively, the IL-4/placebo group received the first dose of recombinant rat IL-4 (PeproTech)/placebo (NaCl) at 0.5 µg/kg intraperitoneally, followed by twice-daily injections for up to seven days. Vehicle animals received an equivalent volume of sterile saline on the same schedule. We opted for using a maximum cumulative dose of 7 µg/kg, distributed on a maximum of 15 single i. p. injections (0.5µg/kg 12 h after the injury and afterwards 0.5µg/kg every 12 h for up to 7 days), based on prior experience from our laboratory and others 19, 23.

Postoperative care included administering buprenorphine (0.05 mg/kg, subcutaneous; Bayer, Leverkusen, Germany) and meloxicam (2 mg/kg, subcutaneous; Boehringer-Ingelheim, Ingelheim, Germany) for pain therapy for five postoperative days. Oral Moxifloxacin was used as an antibiotic prophylaxis (4 mg/kg; Alcon, Fort Worth, TX, USA) for seven postoperative days. Animals also received fluid and nutritional supplements as needed. Manual bladder expression was performed twice daily in SCI animals until autonomous bladder emptying was re-established.

### Neurobehavioral assessment

Functional recovery was assessed using the Basso, Beattie, Bresnahan (BBB) locomotor rating scale 23 at 1, 3, 7, 14, 21, and 28 dpi in all animals. Rats were placed and camera-recorded in an open field for four minutes, and hindlimb locomotor function, joint movement, coordination, and weight-bearing were evaluated by three independent observers blinded to treatment group. Scores (0-21) from all observers were averaged per animal for analysis 24.

Animals also underwent automated quantitative gait analysis using the CatWalk XT system (Noldus Ltd., Wageningen, Netherlands) at 1, 3, 7, 14, 21, 28 dpi 25. Runs were acquired to ensure a minimum of three uninterrupted runs were recorded for each rat at each time point. Paw prints were automatically classified using CatWalk XT software (version 8.1; Noldus Ltd.). In addition, runs were manually verified to correct mislabeling or artifacts by a blinded observer. A total of 195 static and dynamic parameters reflecting fore- and hindlimb function, coordination, speed, and indirect limb strength were analyzed. Group differences were expressed as fold-change ratios between IL-4-treated and vehicle groups. Also, animals without consistent weight-bearing stepping were included in the analysis to capture the full spectrum of (non)-recovery.

At 28 dpi, rats were assessed for erroneous steps using the gridwalk test. Animals traversed a horizontal ladder with irregular spacing in four independent runs. The number of fore- and hindlimb foot placement errors was recorded and post-hoc counted by three blinded observers.

Baseline measurements for the BBB scale and CatWalk XT gait analysis were acquired prior to SCI/sham surgery for all animals.

### Animal perfusion, serum sampling, and tissue processing

At each designated perfusion time point, animals were deeply anesthetized with 4% isoflurane (Baxter, Germany). 1 mL of fresh blood was collected from the right atrium, centrifuged at 1000 g for 5 min at 4 °C, and the serum snap-frozen in liquid nitrogen for later analysis.

Animals were simultaneously transcardially perfused with 50 mL of ice-cold 0.1 M phosphate-buffered saline (PBS; Dulbecco's PBS (1×), Capricorn Scientific, Ebsdorfergrund, Germany), followed by either 150 mL of 4% paraformaldehyde (PFA; in 0.1 M PBS at pH 7.4, Sigma-Aldrich, St. Louis, USA) for immunohistochemistry, or an additional 150 mL of PBS for animals designated for fresh tissue analysis.

Spinal cord tissue was dissected and either post-fixed in 4% PFA for 24 h and cryoprotected in 30% sucrose for 48 h, or snap-frozen for fresh tissue processing. For Immunohistochemistry, segments of 2 cm length, centered at the lesion epicenter, were excised and embedded in tissue embedding medium (Sakura Finetek Europa B.V., Alphen aan den Rijn, Netherlands) on dry ice. Consecutive cross-sections (30 µm) were cut on a cryostat (Leica Biosystems, Nussloch, Germany), dried, and stored at -80 °C until further use.

### Ethics approval for animal experiments

All experimental procedures were approved by the Animal Care Committee of the federal government of Baden-Württemberg, Germany (ethics approval code: G-243/21). All procedures were carried out in accordance with the European Directive 2010/63/EU and approved by the Regierungspräsidium Karlsruhe (35-9185.81/G-269/21).

### Immunohistochemistry

For immunofluorescence staining, fixed spinal cord sections were incubated for 1 h at room temperature in blocking solution containing 1% bovine serum albumin (BSA), 5% non-fat milk powder, and 0.3% Triton-X100 in 0.1 M PBS (all Sigma-Aldrich, St. Louis, MO, USA). Tissue sections were then incubated overnight at 4 °C with the following primary antibodies in the same previously mentioned blocking solution: Anti-NeuN (Host: Rabbit; 1:500; Merck-Millipore, Germany), Anti-APC (Host: Mouse; 1:200; Merck-Millipore, Germany), Anti-GFAP (Host: Rabbit; 1:1,000; Merck-Millipore, Germany), Anti-IBA1 (Host: Mouse; 1:500; Novus Biologicals, USA) for macrophages/microglia, Anti-ARG1 (Host: Rabbit; 1:400; Abcam, UK) or anti-CD206 (Host: Rabbit; 1:2,000; Synaptic Systems, Germany) for M2-macrophages, Anti-iNOS (Host: Rabbit; 1:20; Invitrogen, USA) for M1-macrophages.

Secondary antibodies were diluted in blocking solution without Triton-X100 and applied for 1 h at room temperature: Alexa Fluor 546 goat anti-rabbit (1:500; Thermo Fisher Scientific, USA), Alexa Fluor 568 goat anti-mouse (1:500; Thermo Fisher Scientific, USA) and Alexa Fluor 647 goat anti-rabbit (1:500; Thermo Fisher Scientific, USA). Nuclear counterstaining was performed using DAPI (4′,6-diamidino-2-phenylindole, 1:10,000; Sigma-Aldrich, St. Louis, MO, USA) for 30 min, followed by three washes in PBS. Sections were coverslipped with Mowiol® 4-88 mounting medium (Carl Roth, Karlsruhe, Germany). Isotype controls, consisting of non-specific immunoglobulins at identical concentrations to the primary antibodies, were included in all experiments.

### Imaging analysis

Images were acquired using a confocal laser scanning microscope (LSM 700, Carl-Zeiss, Jena, Germany) at 10× or 20× magnification in 8-bit format, using the tile scan function (scan speed 6, gain 800). Five wavelength channels were used: DAPI 405 nm, Alexa Fluor 546 nm, Alexa Fluor 568 nm, and Alexa Fluor 647 nm, with light transmission ranging from 2.8% to 100%. For quantitative assessment of oligodendrocytes (Adenomatous Polyposis Coli, APC^+^), neurons (NeuN^+^), and macrophages/microglia (Ionized calcium-binding adaptor molecule 1, IBA1^+^, IBA1^+^/ARG1^+^, IBA1^+^/CD206^+^, IBA1^+^/iNOS^+^), semi-automatic cell counting was performed on cross-sections (30 μm) at ± 0 μm, ± 480 μm, ± 1,200 μm, ± 1,920 μm, and ± 2,640 μm from the lesion epicenter. Analyses were conducted independently by three investigators blinded to groups.

Cell counting followed a previously described ImageJ2 (NIH, Bethesda, MD, USA) algorithm 20, 26, 27: Images were split into individual channels, a Gaussian filter (σ = 10.00) was applied to reduce background noise. The IsoData thresholding algorithm was then applied to transform the selected ROI into a binary image. ROIs consisted of the entire spinal cord without the autofluorescence border. Only structures with an area between 50-2000 μm² were included. Then, positive-labelled cells with signals above defined thresholds were counted using the “Analyze Particles” function. Binary images were recombined using the “Image Calculator” function to determine co-stained cell counts. Cell density was expressed as cells/mm².

For assessment of preserved tissue, ImageJ2 was used to process cross-sections stained for GFAP equidistantly from the lesion epicenter (± 0 μm, ± 480 μm, ± 1,200 μm, ± 1,920 μm, and ± 2,640 μm). A ROI was defined around the entire spinal cord in addition to the cystic cavity, with preserved tissue area calculated as the percentage of lesion area from spinal cord area (lesion area / total spinal cord area × 100).

### Transcriptome sequencing

Rat spinal cord tissue stored in in sCelLive® Tissue Preservation Solution was processed by Singleron Biotechnologies GmbH, Germany. Total RNA was isolated using the GeneMAtrix Universal RNA Purification Kit (Roboklon, Germany) and processed according to the manufacturer's protocol for the AccuraCode® RNA-Seq kit (Singleron Biotechnologies GmbH), with the following modifications: Polyadenylated mRNA from each sample was captured using paramagnetic AccuraCode bulk beads (Singleron Biotechnologies GmbH). During reverse transcription, mRNA was converted into cDNA and labelled with sample-specific barcodes and unique molecular identifiers (UMIs). Labelled cDNA was pooled, amplified, and used to construct RNA-seq libraries according to the kit protocol. Libraries were sequenced on an Illumina NovaSeq 6000 platform (paired-end 150 bp) by Macrogen Europe (Amsterdam, Netherlands).

### Differentially expressed genes and pathway enrichment analyses

Differential gene expression analysis was performed using the scanpy.tl.rank_genes_groups() function in Scanpy, based on the Wilcoxon rank sum test with default parameters. Genes expressed above background levels in the majority of samples in either of the compared groups and with an average log₂(Fold Change) > 0.25 were considered for further analysis. Adjusted p-values were computed using the Benjamini-Hochberg correction, with a significance threshold of p_adj < 0.05.

Custom M1 (n = 20 genes) and M2 (n = 17 genes) gene sets were defined for enrichment analysis. Gene set variation analysis (GSVA) was conducted using a Gaussian kernel, and differential activity between IL-4 vs. sham, vehicle vs. sham, and IL-4 vs. vehicle groups was evaluated using limma contrasts with empirical Bayes moderation (eBayes).

To investigate the biological functions of the identified DEGs, Gene Ontology (GO) and Kyoto Encyclopedia of Genes and Genomes (KEGG) enrichment analyses were performed using the clusterProfiler R package (v 3.16.1). Pathways with an adjusted p-value (p_adj) < 0.05 were considered significantly enriched. Selected significant pathways were visualized as bar plots and dot plots.

### Proteomics

Protein samples (≤ 20 µg) were prepared for digestion using the Single Tube Solid Phase Sample Preparation (SP3) method on a KingFisher Apex platform (Thermo Fisher Scientific, 28), with all reagents, solutions, and vessels of high purity and keratin-free. Magnetic bead-based preparation was performed in a 96-well plate format using prototype magnetic bead slurry (Promega, Cat. No. 916738) at a final concentration of 100 µg/µL in a total binding volume of 1000 µL per sample.

Beads were washed three times with dH₂O, then proteins were reduced and alkylated with 10 mM TCEP, 40 mM CAA, and 1% SDS. Samples were incubated at 95 °C for 5 min, followed by 70 °C for 25 min with mixing, and cooled to room temperature. Two µL of bead slurry was added to each sample, ethanol was added to 80% final concentration, and samples were incubated at 24 °C for 20 min with mixing to bind proteins. Beads were washed three times with 1 mL of 80% ethanol and once with 1 mL of 80% acetonitrile (ACN), each wash lasting 4 min with mixing.

Protein digestion was performed with trypsin (1:50 enzyme-to-protein ratio) at 37 °C for 4 h with mixing. Digestion was quenched by adding TFA, and samples were stored overnight at 4 °C. pH was confirmed to be below 2 before StageTip desalting with self-assembled C18 Empore® extraction discs (3M, Maplewood, MN) 29.

Peptides were analyzed on a Vanquish Neo UHPLC system coupled to an Orbitrap Eclipse Tribrid mass spectrometer (Thermo Fisher Scientific). An in-house packed analytical column (75 µm × 200 mm, 1.9 µm ReprosilPur-AQ 120 C18, Dr. Maisch, Germany) was used. Solvent A: 0.1% formic acid; Solvent B: 0.1% formic acid in 80% ACN. Peptides were separated on a 120-min linear gradient: 4% B to 32% B over 100 min, 32% to 49% B over 20 min, followed by a wash at 99% B.

The Orbitrap Eclipse was operated in data-dependent acquisition (DDA) mode 30. MS1: resolution 120,000; AGC target 1.2e6; MaxIT 50 ms; RF Lens 30%; m/z 400-1600; dynamic exclusion 10 s; isolation window centered on most abundant monoisotopic peak; singly charged ions and charge states > 6 excluded; intensity threshold 5e3. MS2: linear ion trap turbo scan rate; AGC target 2.5e4; MaxIT 14 ms; HCD NCE 30%.

Spectra were searched against the UniProt R. norvegicus database (UP000002494) plus a custom contaminant database using Proteome Discoverer 3.1 (Thermo Fisher Scientific) with the Sequest HT search engine. Parameters: fragment mass tolerance 0.6 Da; parent mass tolerance 10 ppm; trypsin enzyme; variable modifications — Oxidation (M), Met-loss (M); fixed modification — Carbamidomethylation (C). Quantification was performed with the precursor ion quantifier node using the Summed Intensity method for protein abundance.

Proteomics data were analyzed in R. Filtering: proteins with < 2 peptides or not designated as master proteins were removed. Data were median normalized to correct for systematic abundance differences. Proteins were retained if quantified in ≥ 2/3 replicates in at least one experimental group. Missing values were imputed in two steps: (1) MNAR values replaced by random draws from a downshifted normal distribution to simulate low-abundance proteins; (2) MAR values imputed using the missForest algorithm 31.

Differential abundance was tested with Student's t-test and Benjamini-Hochberg correction. Proteins with ≥ 2-fold change and p ≤ 0.05 were considered significant. Visualization was performed using tidyverse 32. GO enrichment of significant proteins was performed with ShinyGO 33.

### Serum cytokine analysis

Fresh rat blood samples were centrifuged at 1,000 rpm for 5 min at 4 °C. Serum was separated, snap-frozen in liquid nitrogen, and stored at -80 °C until analysis. Serum levels of GM-CSF, IFN-γ, IL-1α, IL-1β, IL-2, IL-4, IL-5, IL-6, IL-10, IL-12p70, IL-13, IL-17A, IL-17F, IL-18, IL-22, IL-33, KC (CXCL1), MCP-1 (CCL2), and TNF-α were quantified using LEGENDplex™ kits (BioLegend, San Diego, CA, USA; Cat# 740265 and Cat# 76873), according to the manufacturer's instructions. Data acquisition was performed on a BD FACS CANTO II flow cytometer (BD Biosciences, San Diego, CA, USA).

### Human SCI serum analysis

From 2011 onwards, patients with traumatic spinal cord injury (tSCI) treated at a major German trauma center were prospectively included in an institutional registry. Blood samples were collected at the following intervals post-injury: at admission, 4 h, 9 h, 12 h, 1 d, 3 d, 1 w, 2 w, 4 w, 8 w, and 12 w. At each time point, four vials of serum (7.5 mL each) were collected. After a 20-min coagulation period, samples were centrifuged at 3000 rpm, aliquoted, and stored at -80 °C until analysis.

Cytokine assessment was performed using Luminex Performance Human High-Sensitivity Cytokine Panels and ELISA assays 34-36. All quantitative analyses were conducted by a laboratory technician blinded to patient identities and clinical data.

The registry has previously been used for studies aimed at identifying biomarkers for diagnosis and prediction of neurological recovery after SCI 35, 37-39. Although samples were collected prospectively, analyses were conducted retrospectively, and the number of patients per analysis varied depending on sample availability and research focus. To avoid double reporting, each patient was included only once in any given analysis, even if eligible for multiple studies.

Neurological function was graded according to the American Spinal Injury Association (ASIA) Impairment Scale (AIS) at admission and again at 12 weeks post-injury, following the International Standards for Neurological Classification of SCI (ISNCSCI) 40, 41. A favorable outcome was defined as an improvement of at least one AIS grade within 3 months of injury. Initial ISNCSCI assessments were performed within 72 h of admission by the head physical therapist responsible for the patient.

The study was approved by the ethics committee of the local medical faculty (S-514/2011) and by the Landesärztekammer Rheinland-Pfalz (837.188.12/8289-F), Germany.

### Statistical analysis

Group differences were assessed using unpaired two-sample t-tests for cell density measurements in immunohistochemistry analyses; two-way repeated-measures ANOVA followed by Tukey-Kramer post hoc tests for group × timepoint comparisons in neurobehavioral assessments; and one-way ANOVA followed by Bonferroni-corrected t-tests for immunohistochemistry and cytokine data.

Normality was confirmed prior to all parametric tests using the Shapiro-Wilk test. Data are presented as mean ± standard error of the mean (SEM). A p-value < 0.05 was considered statistically significant.

All statistical analyses and visualizations were performed in R (version 4.3.0; R Core Team) 42 within RStudio (version 2023.03.0+386; Posit Software, PBC) and GraphPad Prism (version 9.0; GraphPad Software, San Diego, CA, USA). R packages included ggplot2 for data visualization 43, tidyr for data tidying, and dplyr for data manipulation 44.

## Results

### Intraperitoneal IL-4 treatment enhances locomotor recovery after SCI

**Figure 1:**
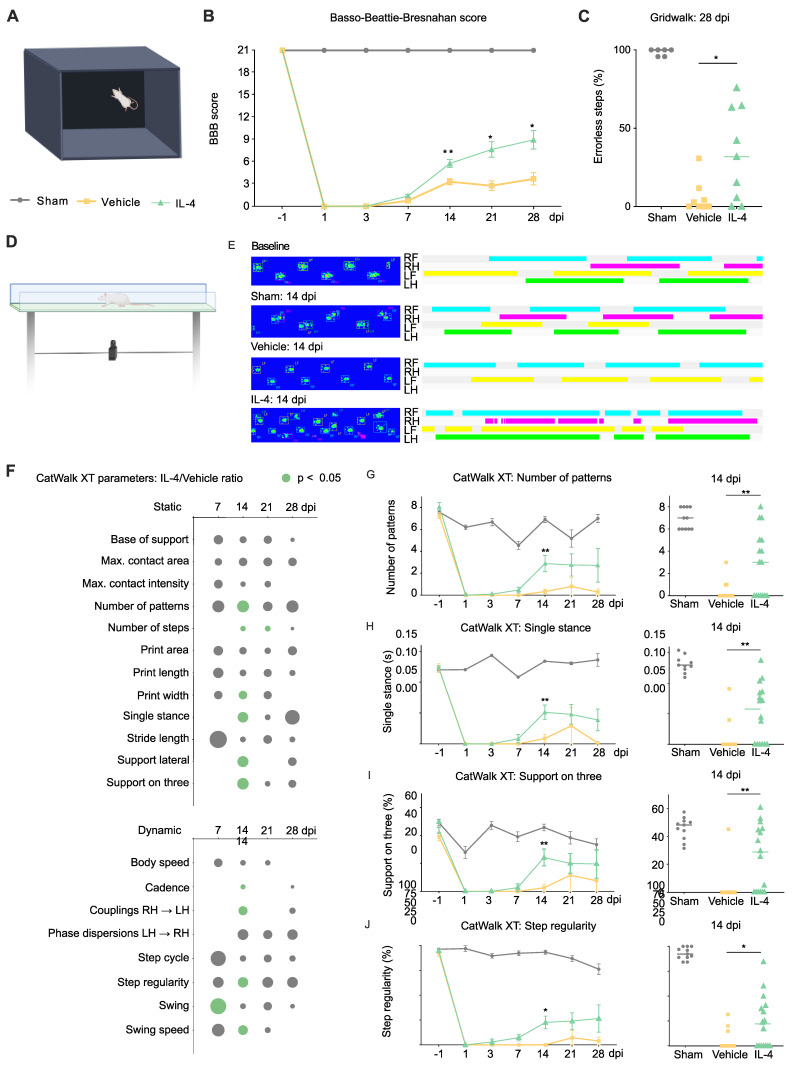
** Systemic IL-4 treatment improves neurological outcome after SCI in rats. (A)** Experimental setup for the open-field Basso, Beattie, Bresnahan (BBB) locomotor rating scale test. **(B)** Longitudinal BBB score profiles for sham-operated rats (blue), SCI and Vehicle (orange), and SCI and IL-4 (green, * p < 0.05, ** p < 0.01; n = 18/group; two-way repeated-measures ANOVA with Tukey's post-hoc). **(C)** Gridwalk test at 28 dpi showing significantly fewer erroneous hindlimb placements in IL-4-treated rats compared to vehicle (n = 9/group; * p < 0.05). **(D)** CatWalk XT gait analysis setup, n=4 runs per time point per rat. **(E)** Representative paw-print profiles from a sham rat, a vehicle rat, and an IL-4-treated rat at baseline and 14 dpi. RF (right forelimb) = turquoise, RH (right hindlimb) = purple, LF (left forelimb) = yellow, LH (left hindlimb) = green. **(F)** Heatmap representation of IL-4/Vehicle fold-changes in selected CatWalk XT parameters over time. Circle size denotes fold-change magnitude; green color indicates statistical significance (p < 0.05, one-way ANOVA with Tukey's post-hoc). **(G-I)** Time courses for number of paw-pattern types per run, single-stance duration, and % time with support on three limbs. IL-4 rats showed significant improvement in all three parameters at 14 dpi (n = 11/group; ** p < 0.01). **(J)** Step regularity index (dynamic parameter) was significantly higher in IL-4-treated rats at 14 dpi (* p < 0.05). Horizontal lines represent mean ± SEM.

Neurological recovery was assessed using a multimodal battery of behavioral tests in IL-4-treated, vehicle-treated, and sham-operated rats (Figure 1A). As expected, sham rats maintained intact hindlimb function (BBB score = 21) throughout the 28-day follow-up. In contrast, SCI induced an immediate and marked reduction in BBB scores on day 1 in both IL-4- and vehicle-treated animals, followed by gradual improvement over time. By 14 dpi, IL-4-treated rats demonstrated significantly greater recovery than vehicle controls (two-way repeated-measures ANOVA with Tukey-Kramer post hoc test, p = 0.0056; Figure 1B), a difference that persisted through day 28 post-injury.

Fine motor coordination assessed by the gridwalk test at 28 dpi revealed a nearly twofold reduction in hindlimb step errors in the IL-4 group compared with vehicle-treated rats (p = 0.0288; Figure 1C).

To complement these rater-based assessments, we applied CatWalk XT automated gait analysis (Figure 1D). Paw print pattern analyses indicated that IL-4 treatment restored hindlimb placement toward sham-like profiles, with significant group differences emerging as early as 7 dpi and peaking at 14 dpi (Figures 1E and F). Improvements remained detectable at 21 and 28 dpi, though with reduced statistical strength.

Static and dynamic gait parameters showed clear benefit from IL-4: Higher paw pattern counts (Figure 1G), increased single-stance contact duration (Figure 1H), greater three-limb support (Figure 1I), all p < 0.01), as well as wider paw prints (Supplementary Figure S2A; p < 0.05) at 14 dpi. Further Dynamic parameters included higher step regularity (p = 0.0138; Figure 1J), faster swing speed, and longer swing duration (Supplementary Figure S2B and C).

Collectively, these results indicate that systemic IL-4 treatment after SCI improves not only the frequency of limb movement but also coordination, regularity, and strength of hindlimb function, with benefits detectable by both observer-rated and automated gait analyses. No direct adverse events due to IL-4 administration were observed in treated rats (also refer to Supplementary Figures S2D and S2E for mortality and weight changes over the course of the treatment).

### Transcriptomic and proteomic characterization of IL-4 therapy after SCI provides mechanistic insight into neuroprotection

**Figure 2:**
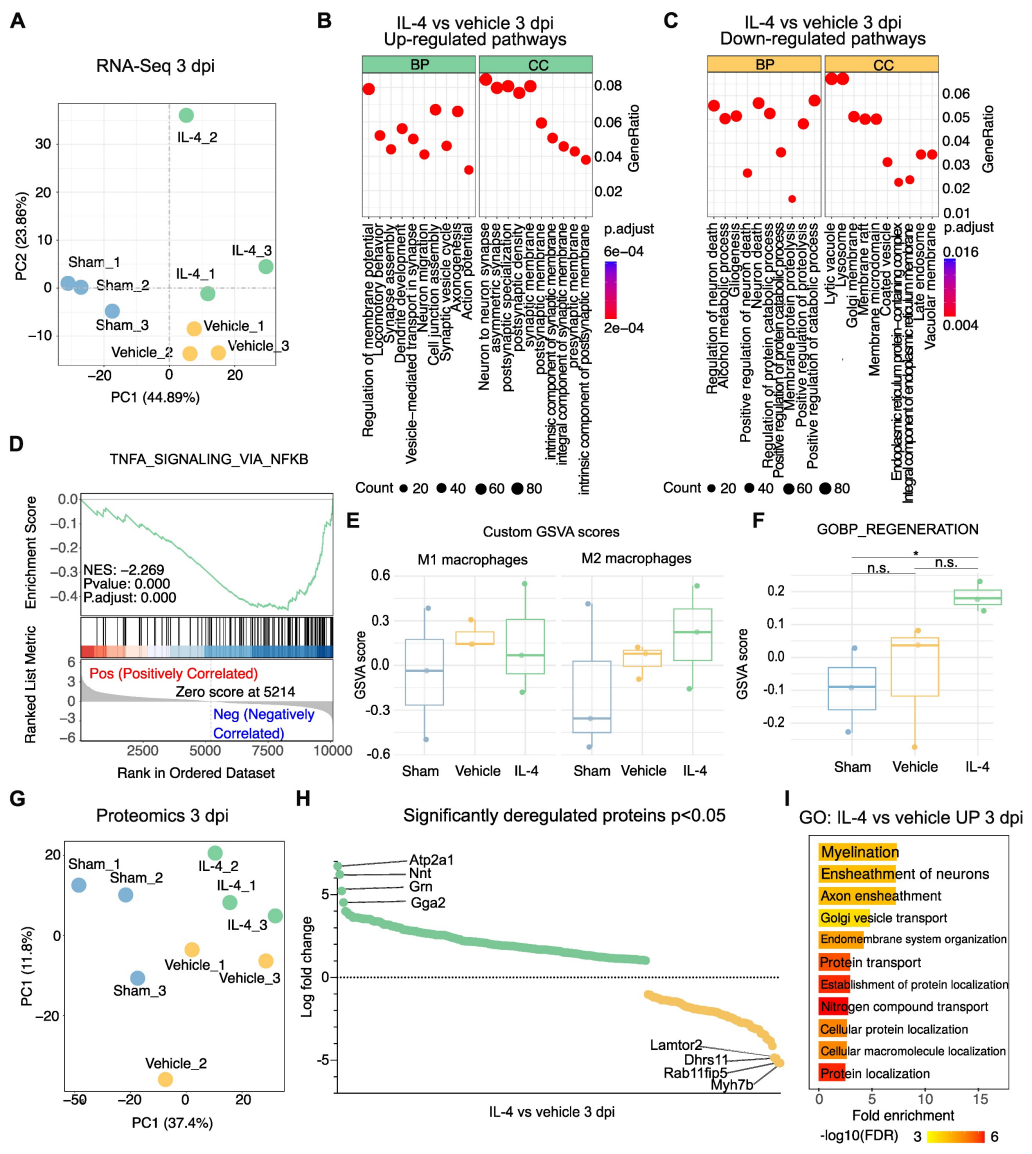
** Transcriptomic and proteomic profiling reveals IL-4-mediated upregulation of axonogenesis and regeneration-associated pathways after SCI. (A)** Principal component analysis (PCA) of bulk RNA sequencing (RNA-seq) data from lesion epicenters at 3 days post-injury (dpi) showing clear separation between sham, SCI + vehicle, and SCI + IL-4 groups (n = 3/group). PC1 reflects injury status, PC2 reflects treatment effect.** (B-C)** Gene ontology (GO) enrichment bubble plots for significantly upregulated (B) and downregulated (C) pathways in IL-4-treated versus vehicle groups at 3 dpi. Enrichment results are categorized by biological process (BP) and cellular component (CC). Bubble size = number of genes in pathway; color = adjusted p-value (Benjamini-Hochberg correction).** (D)** Gene set enrichment analysis (GSEA) plot showing significant negative enrichment of the TNF-α signaling via NF-κB pathway in IL-4-treated samples versus vehicle (normalized enrichment score [NES] = -2.269, adjusted p < 0.001).** (E)** Gene set variation analysis (GSVA) scores for custom-defined M1 macrophage (n = 20 genes) and M2 macrophage (n = 17 genes) signatures in sham, vehicle, and IL-4 groups at 3 dpi. Trends toward reduced M1 and increased M2 scores with IL-4 were not statistically significant.** (F)** GSVA scores for the GO:BP Regeneration gene set showing a strong but non-significant trend toward upregulation in IL-4-treated versus vehicle groups at 3 dpi.** (G)** PCA of label-free quantitative proteomics data from lesion epicenters at 3 dpi (PC1 = 37.4% variance; PC2 = 11.8% variance) demonstrating separation between sham, vehicle, and IL-4 groups.** (H)** Volcano plot of significantly deregulated proteins in IL-4-treated versus vehicle samples at 3 dpi (p < 0.05), with the top four most upregulated proteins highlighted: ATP2A1 (SERCA1), Nnt, Grn (granulin), and Gga2.** (I)** Selected significantly enriched GO pathways (BP category) for upregulated proteins in IL-4 versus vehicle groups at 3 dpi based on proteomic analysis.

To investigate mechanisms underlying the functional improvements observed with IL-4 treatment, bulk RNA sequencing (RNA-Seq) and proteomic analyses were performed on lesion epicenter tissue from sham-operated, vehicle-treated, and IL-4-treated rats at 3 dpi (n = 3 per group; Supplementary Figure S3A). Principal component analysis (PCA) of RNA-Seq data demonstrated clear separation along an SCI-related axis (PC1) and a treatment-related axis (PC2; Figure 2A).

Gene ontology (GO) enrichment of significantly upregulated genes in IL-4-treated rats revealed biological processes (BP) associated with synapse assembly, dendrite development, regeneration and axonogenesis, with corresponding enrichment in cellular components (CC) like neuron-to-neuron synapse (Figure 2B, Supplementary Figures S3B and S3C). Conversely, downregulated pathways included regulation of neuron death and gliogenesis (BP), with reduced lysosomal and adhesion signaling in CC/MF terms (Figure 2C, Supplementary Figure S3D).

Given this reduction in lysosomal signaling, we hypothesized decreased M1 macrophage/microglia activation and polarization toward an M2 phenotype under IL-4 treatment. Gene set enrichment analysis supported this, showing significant downregulation of TNFα-NFκB signaling relative to vehicle (NES = -2.269, adjusted p = 5.2 × 10⁻⁷) as well as significant upregulation of genes pertaining to M2 polarization like *cMYC* (p= 0.0477) and *SMAD1* (p= 0.0367) in IL-4 treated compared to vehicle-treated rats at 3 dpi 45, 46 (Figure 2D, Supplementary Figures S3E and S3F). Gene set variation analysis (GSVA) revealed a non-significant trend toward lower scores for a 20-gene M1-associated set and higher scores for a 17-gene M2-associated set in IL-4 versus vehicle samples (both adjusted p = 0.855; Figure 2E).

GO analysis of significantly upregulated genes also indicated enhanced regeneration signatures in IL-4 versus sham, with non-significant trends over vehicle (Figure 2F). Parallel proteomic profiling corroborated these findings (Figure 2G, Supplementary Figures S3G and S3H). Notably, the most significantly upregulated proteins in IL-4 versus Vehicle included ATP2A1 (SERCA1), Nnt, Grn, and Gga2 (Figure 2H), all implicated in neuroprotection, oxidative stress resistance, and limiting secondary injury 45-50. GO analysis of upregulated proteins showed significant enrichment for myelination and axon ensheathment pathways (Figure 2I).

Collectively, these molecular signatures suggest that systemic IL-4 therapy promotes axonal reorganization, myelin repair, and neuroprotection while attenuating pro-inflammatory signaling at 3 dpi.

### IL-4 treatment limits post-injury cyst size, preserves oligodendrocytes, and promotes M2 macrophage polarization after SCI

**Figure 3:**
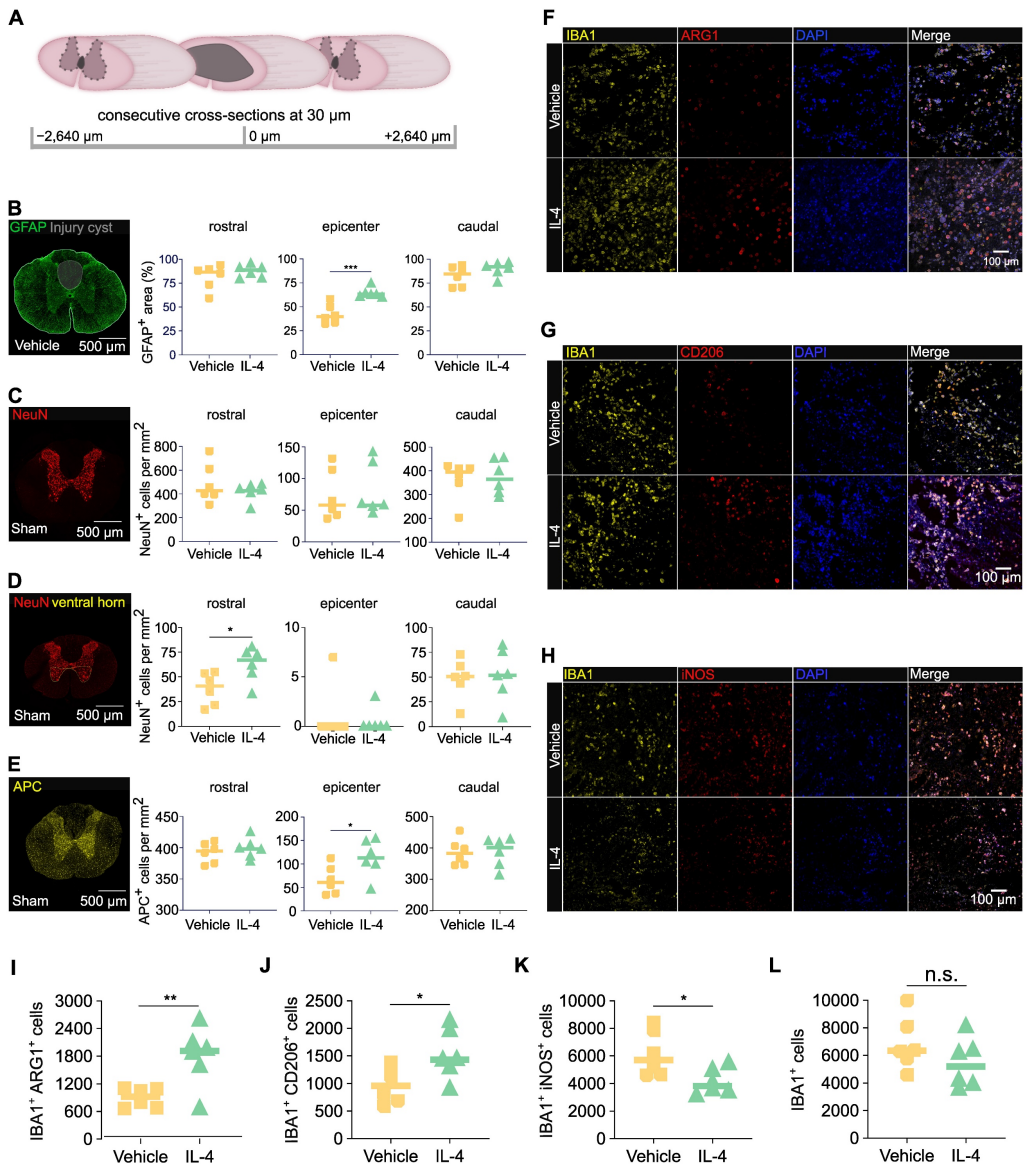
** IL-4 reduces post-injury cyst size, preserves oligodendrocytes, and shifts macrophage/microglia polarization toward an anti-inflammatory phenotype after SCI in rats. (A)** Schematic of consecutive 30-µm cross-sectioning from -2640 µm to +2640 µm relative to the lesion epicenter on spinal cords n=6/group extracted at 28 days post-injury (dpi).** (B)** Representative immunofluorescence (IF) images of fixed spinal cord tissue of vehicle treated rats stained for GFAP (astrocytes) with quantification of GFAP^+^ area as a percentage of total spinal cord cross-sectional area at the epicenter and adjacent levels in IL-4-treated and vehicle-treated SCI rats (n = 6/group, p < 0.001 at epicenter, unpaired Student's t-test). Reduced GFAP^+^ area reflects decreased cyst size and increased preserved tissue under IL-4 treatment.** (C-E)** Representative immunofluorescence (IF) images of post-fixed spinal cord tissue of sham rats stained for NeuN (neurons) and APC (oligodendrocytes) with semi-automatic quantification of NeuN^+^ neurons of the whole spinal cross-section (C), of the ventral horn only (D) and of APC^+^ oligodendrocytes in the whole spinal cord cross-section (E) rostral and caudal to, and at the epicenter of the lesion. IL-4-treated rats show ~2-fold higher oligodendrocyte density than vehicle controls at 28 dpi (p < 0.05), with no significant difference in neuronal density (n = 6/group, scale bar = 500 µm).** (F-G)** Representative IF co-staining at 3 dpi for IBA1 (pan-macrophage marker) with ARG1 (F, M2), CD206 (F, M2), or iNOS (G, M1) in dorsal spinal cord, scale bar = 100 µm. Merged images (including DAPI nuclear stain) highlight double-positive cells. IL-4 treatment increases M2 (IBA1^+^/ARG1^+^ and IBA1^+^/CD206^+^) and reduces M1 (IBA1^+^/iNOS^+^) macrophages compared to vehicle.** (H-L)** Quantification of IBA1^+^ macrophages (H), IBA1^+^/ARG1^+^ (I), IBA1^+^/CD206^+^ (J), and IBA1+/iNOS+ (K) cells, as well as total iNOS^+^ cells (L), at the epicenter (n = 6/group). IL-4 significantly increases M2-macrophage abundance (p < 0.01 for ARG1^+^, p < 0.05 for CD206^+^) and decreases M1-macrophages (p < 0.05). Statistical analysis: unpaired Student's t-test.

To assess structural preservation, cross-sections of spinal cord tissue were collected at 28 dpi from IL-4 and vehicle-treated rats. Regions analyzed included the lesion epicenter and segments 2640 μm rostral and caudal (Figure 3A). Staining for GFAP (astrocytes/astrogliosis) followed by GFAP-based volumetric analysis revealed significantly smaller cystic cavities and hence a higher amount of preserved tissue at the epicenter in post-fixed spinal cords of rats receiving the IL-4 treatment compared with vehicle-treated controls (Figure 3B; unpaired t-test). Although NeuN⁺ neuron counts were not significantly different in post-fixed tissue between IL-4 and vehicle treated rats across all levels (cranial, epicenter and caudal; Figure 3C), quantification of NeuN⁺ cells revealed a significantly higher count of neurons in IL-4 treated compared to vehicle rats in the ventral horn of post-fixed spinal cord tissue caudal to the lesion (Figure 3D; no difference in cranial spinal cord tissue or epicenter), revealing more pronounced neuronal preservation caudal to the lesion under IL-4 treatment (Figure 3D). APC⁺ oligodendrocyte density was approximately two-fold higher in IL-4-treated compared to vehicle rats at the epicenter (p = 0.047; Figure 3E). No significant differences were observed in rostral or caudal regions for APC^+^ cells.

To validate early immune modulation suggested by omics data, we assessed macrophage polarization at 3 dpi via co-staining for IBA1 (pan-macrophage/microglia = myeloid cell marker) with ARG1 (M2), CD206 (M2), or iNOS (M1). IL-4 treatment significantly increased densities of IBA1⁺/ARG1⁺ and IBA1⁺/CD206⁺ M2 macrophage/microglia abundance (Figures 3F-G, 3I-J) and reduced IBA1⁺/iNOS⁺ M1 macrophage/microglia abundance (Figures 3H, 3K) at the epicenter versus vehicle (all p < 0.05; unpaired t-tests). Total IBA1⁺ macrophage/microglia density was modestly lower with IL-4, though not statistically significant (Figure 3L).

These results indicate that systemic IL-4 limits glial post-injury cyst size, preserves oligodendrocytes, and promotes an anti-inflammatory M2 myeloid phenotype in the acute post-injury phase, supporting the molecular findings.

### Systemic pro-inflammatory immune response after SCI is attenuated by IL-4 in rats and associates with functional outcomes in patients

**Figure 4:**
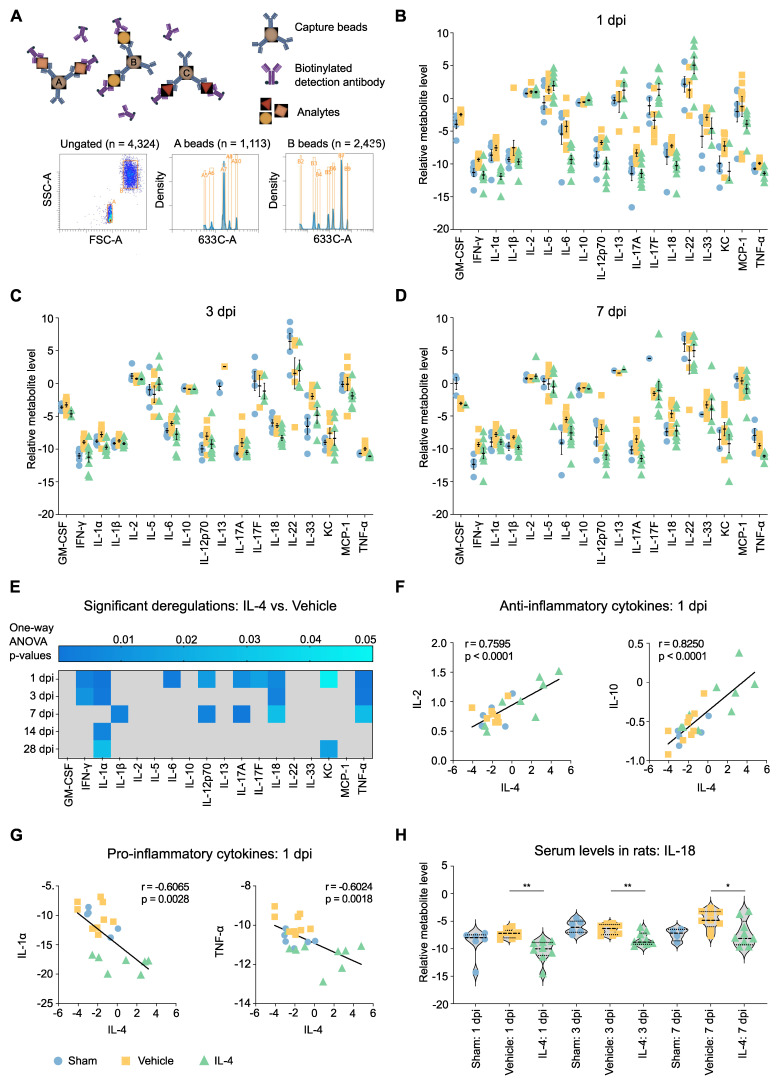
** IL-4 attenuates the systemic pro-inflammatory cytokine response after SCI in rats. (A)** Schematic of bead-based flow cytometry (FC) assay used for high-throughput seromics. FSC-A = forward scatter area; SSC-A = side scatter area. **(B-D)** Normalized serum concentrations of 18 cytokines at 1 dpi (B), 3 dpi (C), and 7 dpi (D) in sham (blue), SCI vehicle-treated (orange), and SCI IL-4-treated (green) rats (n = 9 per group, note that not all samples showed measurable concentrations in all cytokines). SCI induces a global increase in pro-inflammatory cytokines in vehicle-treated animals, largely mitigated by IL-4 treatment. Data shown as mean ± SEM. **(E)** Fold-change (IL-4 vs. Vehicle) in serum cytokine levels at all time points after SCI, with significance indicated by shading (blue intensity = adjusted p value from ANOVA with Tukey's post hoc correction).** (F-G)** Pearson correlations between serum IL-4 levels at 1 dpi and anti-inflammatory cytokines IL-2 and IL-10 (F) or pro-inflammatory cytokines IL-1α and TNF-α (G).** (H)** Violin plots of serum IL-18 concentrations at 1, 3, and 7 dpi in sham, vehicle, and IL-4 groups. IL-4 treatment significantly reduced IL-18 at all time points (p < 0.01, p < 0.05; unpaired Student's t-test).

Given the observed local neuroprotective effects of IL-4 on spinal cord tissue and macrophage/microglia polarization, we next examined whether systemic administration also modulates the circulating cytokine milieu after SCI. Serum from IL-4-treated, vehicle-treated, and sham rats was analyzed using a flow cytometry-based bead assay profiling 18 cytokines (Figure 4A).

Vehicle-treated rats exhibited robust increases in multiple pro-inflammatory cytokines at 1, 3, and 7 dpi relative to sham, confirming the sensitivity of this high-throughput seromics approach (Figures 4B, 4C and 4D). These changes were found significant for GM-CSF, INF- γ, IL-1β, IL-17A, IL-33 and TNF-α (Supplementary Figure S4B). Cytokine elevations diminished by 14 and 28 dpi (Supplementary Figures S4C and S4D). In contrast, IL-4 treatment significantly suppressed many of these early pro-inflammatory responses, with the largest differences observed at 1 and 7 dpi, suggesting a biphasic inflammatory profile after SCI (Figure 4E).

Serum IL-4 levels at 1 dpi were positively correlated with anti-inflammatory cytokines such as IL-2 and IL-10 and negatively correlated with pro-inflammatory mediators including IL-1α and TNF-α (Figure 4G, Supplementary Figures S5A, S5B and S5C), supporting a systemic immunomodulatory role of IL-4. Notably, IL-18 and TNF-α reductions were consistent across 1, 3, and 7 dpi, whereas IL-12p70 and IL-17A displayed bimodal suppression patterns. IL-18 suppression is of particular interest, given its established role in post-SCI allodynia (Figure 4H, Supplementary Figure S5D) 47.

### Translation to clinical data

To explore the translational relevance of these systemic changes, we compared the rat seromics results to previously published longitudinal cytokine data from SCI patients 48. The human dataset included patients dichotomized by functional improvement, defined as ≥1 AIS grade increase at 3 months post-injury.

**Figure 5:**
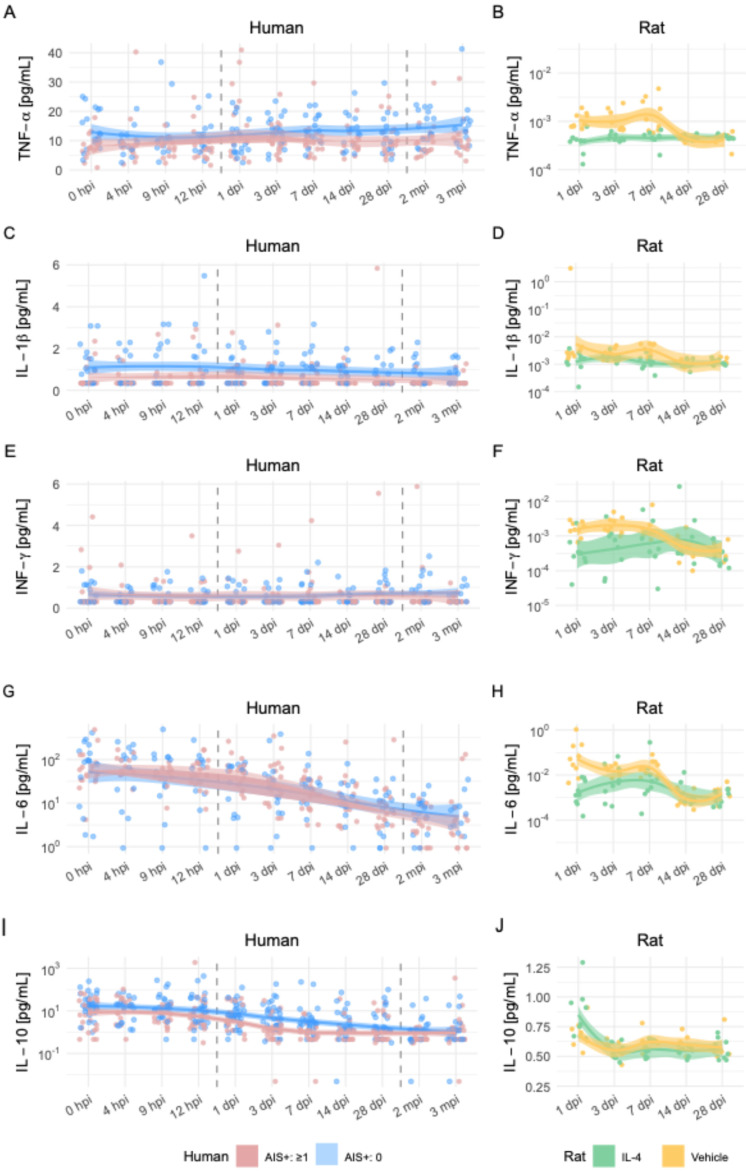
** Comparative time-series analysis of cytokine dynamics after SCI in humans and rats.** Longitudinal profiles of five cytokines in human SCI patients (left column) and rat SCI models (right column) over matched post-injury periods.** (A, B)** Tumor necrosis factor-α (TNF-α), **(C, D)** interleukin-1β (IL-1β), **(E, F)** interferon-γ (IFN-γ), **(G, H)** interleukin-6 (IL-6), and **(I, J)** interleukin-10 (IL-10). For human data, time points range from admission (0 hpi) to 3 months post-injury (mpi), and patients are stratified into those with AIS (American Spinal Injury Association Impairment Scale) improvement ≥1 point (green) versus no improvement (blue). For rat data, measurements span 1 to 28 days post-injury (dpi), with groups indicated as IL-4-treated (red), vehicle-treated SCI (yellow), and sham (grey). Dots represent individual measurements; smoothed LOESS curves with 95% confidence intervals illustrate temporal trends. Human data reflect serum cytokine levels obtained via Luminex or ELISA, while rat data derive from bead-based multiplex flow cytometry.

Patterns of cytokine regulation showed both convergences and divergences between species:

- **TNF-α**: Higher levels in improved patients during the first week post-injury; in rats, TNF-α was suppressed by IL-4 at 3 dpi, with time-dependent variability (Figures 5A-D).
- **IFN-γ**: Improved patients had slightly lower levels; IL-4-treated rats showed reduced IFN-γ in the first week, with rebound after cessation of treatment (Figures 5E-F).
- **IL-6**: In patients, levels decreased over time, with slightly higher values in the improved group; in rats, IL-4 treatment increased IL-6 peaking at 7 dpi, suggesting possible benefit of subacute IL-6 elevation (Figures 5G-H).
- **IL-10**: Lower in improved patients during 7-28 days; similarly reduced in IL-4-treated rats with better functional recovery (Figures 5I-J).

These cross-species observations support a model in which IL-4-mediated modulation of specific pro- and anti-inflammatory cytokines—particularly suppression of IL-18, TNF-α, and IFN-γ—may contribute to improved neurological outcomes after SCI. The data highlight the complexity and strong time dependence of cytokine effects, underscoring the need for temporally targeted therapeutic strategies.

## Discussion

Traumatic spinal cord injury (SCI) is a catastrophic event that imposes severe lifelong disability, with an increasing incidence and substantial personal and socioeconomic burden worldwide 49, 50. Current acute management strategies, including prompt decompressive surgery with vertebral stabilization and optimized medical care, primarily aim to limit further injury but offer limited neuroprotective or regenerative benefits 56-58. Consequently, there remains an urgent need for adjunctive therapies targeting the secondary injury cascade to improve functional recovery 51-53.

In the present study, we demonstrate that repeated systemic administration of IL-4 after thoracic clip-contusion SCI significantly improves functional recovery in rats, as assessed by a multimodal battery of neurobehavioral tests. Mechanistically, transcriptomic and proteomic profiling revealed activation of axonogenesis and wound-healing pathways, suppression of neuronal death and pro-inflammatory signaling, and preservation of oligodendrocytes, in association with M2 macrophage/microglia polarization and reduced post-trauma cyst size. Complementary high-throughput seromics identified a robust post-SCI pro-inflammatory cytokine response that was attenuated by IL-4. Importantly, when mapped to longitudinal cytokine profiles in SCI patients, suppression of specific pro-inflammatory mediators was associated with favorable neurological outcomes, underscoring the translational potential of systemic IL-4 immunomodulation.

### Targeting post-SCI inflammation: from rationale to systemic immunomodulation

The dual nature of the inflammatory response after SCI is well recognized: while essential for debris clearance and initiating repair, unrestrained inflammation exacerbates secondary injury via oxidative stress, excitotoxicity, and glial scar formation 54, 55. Modulating this response toward a reparative phenotype has therefore emerged as a major therapeutic goal 56, 57. Clinically, corticosteroid therapy - most notably methylprednisolone - remains one of the most extensively examined immunomodulatory interventions. However, due to its modest efficacy and potential adverse effects and data generated regarding its utility in SCI, current guidelines do not recommend its application in SCI patients 58-61. This warrants a window of opportunity for the use of a more targeted immune-modulatory approach.

IL-4 is a pleiotropic immunomodulatory cytokine capable of inducing CNS-resident and infiltrating myeloid cells toward an anti-inflammatory, pro-regenerative M2 phenotype 62, 63. Preclinical studies have shown that both intrathecal and systemic IL-4 delivery can enhance M2 polarization and improve histological and molecular markers of repair after SCI 18, 19. However, earlier systemic regimens reported functional recovery only at a phenotypic level 18, while intrathecal delivery - although effective - may be less suitable for achieving systemic immune modulation. Our approach combined repeated systemic dosing with a higher IL-4 concentration (0.5 µg/kg vs. 0.35 µg/kg in prior work 19), achieving robust behavioral improvement and revealing mechanistic correlates at both the tissue and systemic levels.

### Behavioral improvement: beyond frequency of movement

While the BBB locomotor score remains the most widely used index of gross motor function after experimental SCI, it is limited in detecting subtle changes in coordination and gait dynamics. By integrating BBB scoring with CatWalk XT automated gait analysis and the Gridwalk test, we captured a richer functional profile. Notably, IL-4-treated rats exhibited earlier and more consistent improvements in coordination, interlimb regularity, and weight support, with differences apparent as early as 14 dpi in several parameters. These findings suggest that IL-4's benefits extend beyond basic locomotor recovery to include fine motor control, likely reflecting preservation of spinal cord pathways subserving proprioception and coordination.

### Local effects: neuroprotection, oligodendrocyte preservation, and reduced post-injury cyst size

Immunohistochemistry at 28 dpi revealed reduced cyst volume and greater tissue preservation in IL-4-treated rats, accompanied by significantly higher APC+ oligodendrocyte density but no significant NeuN+ neuronal preservation relative to vehicle controls. While the lack of neuronal rescue may reflect the irreversibility of acute neuronal loss, oligodendrocyte survival is critically important for remyelination and conduction recovery. The observed reduction in CXCL1, a chemokine known to inhibit oligodendrocyte migration 64, may contribute to this effect. Corroborating this, a recent study used microparticles to deliver IL-4, IL-10, and IL-13 into the injury site after SCI and revealed significantly lower spinal cord atrophy compared to controls 4.

Mechanistically, this IL-4 pro-regenerative effect appears to be starkly macrophage-polarization mediated. Indeed, an increased M2 macrophage predominance (IBA1+/ARG1+ and IBA1+/CD206+) and reduced M1 macrophage abundance (IBA1+/iNOS+) were observed at 3 dpi, attenuating glial scarring, and facilitating a regenerative microenvironment. Upregulation of granulin - a factor implicated in lysosomal clearance of myelin debris 65 —and other proteins linked to axonal reorganization on a transcriptomic level aligns with the observed tissue preservation and functional recovery. Interestingly, a study used lipid nanoparticles (LNPs) to deliver Mms6 mRNA into macrophages and hence improved locomotor functional recovery after traumatic SCI in mice through abrogating anti-ferroptosis, which is another promising avenue of non-cell-based therapy 66.

### Further transcriptomic and proteomic insights

Bulk RNA sequencing and proteomics at 3 dpi provided converging evidence for IL-4-mediated neuroprotection. Enriched pathways included synapse assembly, dendrite development, axonogenesis, and myelination, alongside suppression of neuron death, gliogenesis, and TNFα-NFκB signaling, in addition to enhanced oxidative stress resilience, M2 polarization and improved regeneration, consistent with the observed preservation of neurons and oligodendrocytes. Proteomic upregulation of SERCA1 (ATP2A1), granulin, Nnt, and Gga2 suggests potential mitigation of neuropathic pain and oxidative stress, in addition to structural repair 67-71. The time point for transcriptomic and proteomic analyses (3 dpi) was reasoned to capture early subacute molecular changes associated with IL-4-mediated immunomodulation, which precedes the functional improvements observed at 7 dpi and could hence delineate the underlying mechanisms for this improvement. These findings support the role of IL-4 treatment in promoting axonal remodeling, myelin repair, and structural integrity, which in turn are key determinants of functional restoration.

### Systemic effects and translational relevance

Our high-throughput bead-based cytokine profiling revealed a robust systemic pro-inflammatory response in vehicle-treated rats during the first week post-SCI, with IL-4 significantly suppressing key mediators such as IL-18, TNF-α, and IFN-γ. The biphasic cytokine modulation pattern—early (1 dpi) and subacute (7 dpi)—suggests IL-4 influences multiple inflammatory phases. IL-18 suppression is notable given its established role in neuropathic pain development after SCI 47.

When compared to human SCI serum data, lower post-injury TNF-α and IFN-γ levels correlated with better functional outcomes, consistent with the cytokine patterns observed in IL-4-treated rats. IL-6 and IL-10 displayed more complex, time-dependent relationships, highlighting the need for temporally optimized immunomodulation. The observations made in both species show a biphasic regulation of serum cytokines after SCI, indicative of a potentially more promising therapeutic potential of temporally tuned immunomodulation rather than indiscriminate immune suppression 72. Correlating cytokine levels with patient outcomes enabled clinical contextualization of the IL-4-induced reduction of pro-inflammatory mediators observed in rats 48, 73. The concordance of specific cytokine changes across species strengthens the translational rationale for systemic IL-4 therapy.

Previous reports have indicated potential adverse effects of IL-4, such as pathological fibrosis, atherosclerosis, and exacerbation of allergic conditions like asthma, raising concerns regarding its clinical applicability 74, 75. However, previous clinical trials involving IL-4 administration in cancer patients have dispersed some of these concerns by demonstrating an acceptable safety profile 76-78.

### Potential mechanisms beyond inflammation control

Improved general condition and weight profiles in IL-4-treated rats suggest possible systemic benefits, potentially via stabilization of cardiovascular or metabolic parameters—factors known to influence SCI outcomes 79. Preservation of dorsal white matter oligodendrocytes may support proprioceptive pathway integrity, contributing to better coordination. Furthermore, IL-4's suppression of pro-inflammatory cytokines implicated in neuropathic pain pathways 80-82 may indirectly enhance functional recovery by reducing pain-related motor inhibition, especially with M2-macrophage/microglia polarization known to mediate pain relief 45.

### Limitations

While this study integrates multi-modal analyses to provide a comprehensive mechanistic picture, several limitations remain. We did not assess IL-4's effects on systemic lymphoid organs or specific circulating immune cell populations, which could yield additional mechanistic insights. The cytokine assays did not distinguish endogenous from exogenous IL-4, though strong correlations with downstream anti-inflammatory mediators support bioactivity. Finally, while our human data analysis supports translational relevance, it remains retrospective and limited to cytokines overlapping with the rat panel. In addition, we did not explore other delivery strategies that have proven effective in targeted drug delivery for SCI therapy, such as nanoparticles 83. Prospective clinical studies will be essential to confirm IL-4's therapeutic potential and optimize dosing, timing, and route of administration in humans. A direct comparison between high- and low-dose IL-4 groups was not performed and would be valuable in establishing a dose-response relationship for IL-4 in SCI. This study was designed based on the hypothesis that a higher IL-4 dose than previously tested would not only replicate histological benefits but also yield clearer functional improvements—an effect that we indeed observed 18, 19. Whether prolonged IL-4 exposure enhances long-term recovery or risks overmodulating the immune system remains to be determined. We acknowledge that integrative transcriptomic and proteomic analyses across multiple time points (1 dpi, 3 dpi, 7 dpi) would establish a more elaborate temporal resolution and strengthen their correlation with the functional outcomes surveyed in rats. Also, when assessing tissue scarring, we only provided analyses of post-injury cyst size using GFAP staining. This could have been improved by adding additional markers such as CSPGs or NG2. Lastly, only female rats were used in this study, potentially reducing gender as a confounder but possibly limiting the generalizability of our findings.

## Conclusions

In summary, our study demonstrates that systemic IL-4 treatment after SCI improves locomotor recovery and coordination in rats, underpinned by a distinct mechanistic profile of local and systemic immunomodulation. By promoting M2 macrophage/microglia polarization, preserving oligodendrocytes, attenuating post-injury cyst size, and activating transcriptomic and proteomic programs associated with axonogenesis and neuroprotection, IL-4 shifts the post-injury environment toward repair. High throughput seromics further revealed suppression of key pro-inflammatory cytokines in both acute and subacute phases, with patterns that aligned with improved outcomes in SCI patients. These findings not only extend previous IL-4 studies by demonstrating functional benefit alongside mechanistic insight, but also highlight IL-4 as a promising candidate for systemic, temporally targeted immunomodulatory therapy in acute SCI.

## Supplementary Material

Supplementary figures and table legends.

Supplementary tables.

## Data Availability

The mass spectrometry proteomics data have been deposited to the ProteomeXchange Consortium via the PRIDE partner repository with the dataset identifier PXD065980. The RNA-Seq dataset of IL-4 vs vehicle-treated sample is available under the accession numbers GSE303532 and GSM9128573 on the Gene Ontology Omnibus (GEO) repository.

## Abbreviations

ACN: acetonitrile
AIS: ASIA Impairment Scale
APC: adenomatous polyposis coli
ARG1: arginase-1
ASIA: American Spinal Injury Association
BBB: Basso, Beattie, Bresnahan locomotor rating scale
BP: biological process
CAA: chloroacetamide
CC: cellular component
CCL2: chemokine (C-C motif) ligand 2
CNS: central nervous system
CXCL1 (KC): chemokine (C-X-C motif) ligand 1
DAPI: 4′,6-diamidino-2-phenylindole
DDA: data-dependent acquisition
DEGs: differentially expressed genes
dpi: days post-injury
dH₂O: distilled water
DNA: deoxyribonucleic acid
ELISA: enzyme-linked immunosorbent assay
FACS: fluorescence-activated cell sorting
GFAP: glial fibrillary acidic protein
GO: gene ontology
GSVA: gene set variation analysis
HCD: higher-energy collisional dissociation
IBA1: ionized calcium binding adaptor molecule 1
IFN-γ: interferon-gamma
IL-4: interleukin-4
IL-6: interleukin-6
iNOS: inducible nitric oxide synthase
ISNCSCI: International Standards for Neurological Classification of SCI
KEGG: Kyoto Encyclopedia of Genes and Genomes
LC-MS/MS: liquid chromatography-tandem mass spectrometry
LED: light emitting diode
MF: molecular function
MCP-1: monocyte chemoattractant protein-1
MNAR: missing not at random
MS1/MS2: mass spectrometry level 1/2
NES: normalized enrichment score
NFκB: nuclear factor kappa-light-chain-enhancer of activated B cells
NeuN: neuronal nuclei
Nnt: nicotinamide nucleotide transhydrogenase
O₂: oxygen
PCA: principal component analysis
PBS: phosphate-buffered saline
PFA: paraformaldehyde
RNA-Seq: RNA sequencing
ROI: region of interest
SCI: spinal cord injury
SEM: standard error of the mean
SDS: sodium dodecyl sulfate
SP3: single-pot, solid-phase-enhanced sample preparation
TBI: traumatic brain injury
TCEP: tris(2-carboxyethyl)phosphine
TNF-α: tumor necrosis factor-alpha
UHPLC: ultra-high-performance liquid chromatography
UMI: unique molecular identifier
