# Supplementary material for: Systemic Interleukin-4 Application Promotes Functional Recovery and Reprograms Neuroinflammatory and Molecular Responses after Spinal Cord Injury in Rats
Source: Theranostics. 2026 Feb 11;16(9):4726–44. doi: 10.7150/thno.123815 (PMC12964225; doi:10.7150/thno.123815)

Supplementary Files: Systemic Interleukin-4 application promotes functional recovery and reprograms neuroinflammatory and molecular responses after spinal cord injury in rats.

Obada T. Alhalabi[1,2]*, Stefan Heene[1,2]*, Guoli Zheng[1,2], Raban Heller[3,4,5], Tim Schubert[6], Marcin Luzarowski[7], Xaiowei Zha[1,2], Johannes Walter[1,2], Lea Hansen-Palmus[8], Bahram Biglari[9], Xing-Jin Wang[1,2], Laura Ruebenacker[1,2], Thomas Skutella[10], Karl Kiening[1,2], Christian P. Schaaf[6], Sandro M. Krieg[1,2], Andreas W. Unterberg, Klaus Zweckberger[1,11], Alexander Younsi[1,2]

[1]Department of Neurosurgery, Heidelberg University Hospital, Heidelberg, Germany
[2]Medical Faculty, Heidelberg University, Heidelberg, Germany
[3]Institute for Experimental Endocrinology, Charité—Universitätsmedizin Berlin, corporate member of Freie Universität Berlin, Humboldt-Universität zu Berlin, Berlin Institute of Health, Berlin, Germany
[4]Department of Food Chemistry and Toxicology, Technische Universität Berlin, Berlin, Germany
[5]Bundeswehr Hospital Berlin, Department of Traumatology and Orthopaedics, Septic and Reconstructive Surgery, Berlin, Germany
[6]Institute of Human Genetics, Heidelberg University, Heidelberg, Germany.
[7]Core Facility for Mass Spectrometry and Proteomics, Center for Molecular Biology at Heidelberg University (ZMBH), Heidelberg, Germany.
[8]Institute of Virology, Technical University of Munich, Helmholtz Munich, Munich, Germany
[9]Department of Paraplegiology, BG Trauma Centre Ludwigshafen, 67071 Ludwigshafen, Germany
[10]Department of Neuroanatomy, Institute for Anatomy and Cell Biology, University of Heidelberg, Heidelberg, Germany
[11]Department of Neurosurgery, Brunswick City Hospital, Brunswick, Germany

*contributed equally

Correspondence:
Alexander Younsi, MD
Department of Neurosurgery,
Heidelberg University Hospital,
Im Neuenheimer Feld 400
69120 Heidelberg, Germany
alexander.younsi@med.uni-heidelberg.de

Supplementary Figure S1. Overview of experimental design, treatment allocation, and analytical workflow for IL-4 application after SCI in rats. (A) Timeline of experimental procedures. Rats underwent a T10 laminectomy and were randomized to either SCI (clip-compression with 28 g for 60 s) or sham. SCI rats were further randomized to receive intraperitoneal IL-4 or saline (vehicle) injections for up to 7 days post-SCI. All animals received postoperative care including antibiotics, analgesics, and regular weight monitoring. At 1, 3, 7, 14, 21, and 28 days post-injury (dpi), behavioural testing, serum sampling, and/or perfusion were performed (behavioural tests only at 21 dpi). The final time point was 28 dpi. (B) Overview of analytical methods used in the study, including behavioural assessments [Basso, Beattie, Bresnahan (BBB) locomotor rating scale, Gridwalk test, and CatWalk XT gait analysis], serum cytokine profiling via bead-based multiplex flow cytometry, and immunohistochemical analysis of extracted spinal cords.

Supplementary Figure S2. Mortality, weight course, and CatWalk XT gait analysis parameters indicate improved functional recovery in IL-4–treated rats after SCI. (A–C) Time profiles for selected CatWalk XT gait analysis parameters in vehicle and IL-4–treated SCI rats: (A) print width (cm), (B) swing time (s), and (C) swing speed (cm/s). Left panels show longitudinal changes; right panels present statistical comparisons at key time points. IL-4–treated rats demonstrated significantly better recovery for all three parameters at 7 and 14 dpi ($p < 0.01$, ANOVA with Tukey's post hoc correction). (D) Mortality across all experimental groups throughout the study. Deceased rats were replaced to maintain group sizes; final numbers are indicated in each bar. dpi = days post-injury. (E) Body weight trajectory (grams) over the course of the experiment; day −1 represents the pre-surgery baseline. IL-4–treated rats showed better weight recovery compared to vehicle-treated rats, with a significant difference at day 5 (n = 18 per group; * $p < 0.05$).

Supplementary Figure S3. Transcriptomic and proteomic profiling of spinal cord tissue reveals IL-4–mediated molecular signatures at 3 dpi. (A) Experimental overview of matched transcriptomic (RNA-Seq) and corresponding proteomic profiling from hemi-sections of spinal cord tissue collected 3 days post-injury (dpi) in IL-4–treated, vehicle-treated, and sham-operated rats (n = 3 per group). m/z = mass-to-charge ratio. (B) Volcano plot of genes in the GO:BP REGENERATION set, comparing IL-4–treated spinal cords to vehicle controls at 3 dpi. Data are shown as $\log_2$ fold-change versus $-\log_{10}$ p-value; genes with a false discovery rate (FDR) < 0.05 are highlighted. (C) Network plot showing significantly enriched Gene Ontology (GO) terms (biological process, cellular component, molecular function) upregulated in IL-4 versus vehicle at 3 dpi, based on Kyoto Encyclopedia of Genes and Genomes (KEGG) analysis. Only terms with adjusted $p < 0.05$ are displayed. (D) Network plot showing significantly enriched GO terms downregulated in IL-4 versus vehicle at 3 dpi (adjusted $p < 0.05$). (E) Counts per million (CMP)-normalized Expression levels of genes associated with M2-polaritazation in sham, vehicle, and IL-4 treated rats (n=3 each) at 3 dpi, depicted are significant p-values (unpaired t-test). (F) Summary table of top significantly downregulated Hallmark pathways from gene set enrichment analysis (GSEA), with normalized enrichment score (NES), nominal p-value, and adjusted p-value (padj) indicated. (G) Total number of identified peptides across biological replicates in each group (n = 3 per group). (H) Total number of identified proteins after filtering across biological replicates in each group (n = 3 per group).

Supplementary Figure S4. Serum cytokine levels indicate sustained attenuation of inflammation by IL-4 treatment. (A) Representative immunofluorescence (IF) images of post-fixed spinal cord tissue of sham rats stained for $NeuN^+$ neurons in the whole cross-section and ventral horn of vehicle rats (B ANOVA (Analysis of Variance) with Tukey's post hoc correction output of serum cytokines (Sham vs. Vehicle) from every time point after SCI of the study, with significance indicated by color (blue to turquoise intensity = adjusted p value). (C) Serum levels of 18 cytokines at 14 dpi in sham (grey), SCI vehicle-treated (yellow), and SCI IL-4–treated (red) rats. (D) Serum cytokine levels at 28 dpi in the same groups. In the post-acute period, a general decline in pro-inflammatory cytokine levels was observed, including in vehicle-treated SCI rats. Error bars represent standard error of the mean (SEM).

Supplementary Figure S5. IL-4 serum levels and cytokine correlations support systemic anti-inflammatory modulation after SCI. (A) Serum IL-4 levels in sham, vehicle, and IL-4–treated rats at 1 dpi (*$p < 0.05$, ANOVA). (B) Correlation analysis of IL-4 levels with anti-inflammatory cytokines IL-5 and IL-13 at 1 dpi. (C) Correlation analysis of IL-4 levels with pro-inflammatory cytokines IFN-γ, IL-6, IL-12p70, IL-18, IL-33, and MCP-1 at 1 dpi. (D) Violin plots showing serum levels of IFN-γ, IL-1α, IL-12p70, IL-17A, KC (CXCL1), and TNF-α in sham, vehicle, and IL-4–treated rats at 1, 3, and 7 dpi. All listed cytokines showed significantly lower levels under IL-4 treatment at all time points (**$p < 0.01$, *$p < 0.05$).

Supplementary Table S1. RNA-sequencing gene expression matrix (counts per million, CPM) for all samples in IL-4 (n=3), vehicle (n=3), and sham (n=3) groups.

Supplementary Table S2. Differentially expressed genes (DEGs) from RNA-sequencing analysis, including fold change values for IL-4 (n=3) versus vehicle (n=3) samples.

Supplementary Table S3. Gene set variation analysis (GSVA) scores for custom gene sets and selected Gene Ontology pathways.

Supplementary Table S4. Statistical results for differentially expressed proteins identified after IL-4 treatment compared to vehicle, including fold change, p-values, and false discovery rate (FDR).

Supplementary Figure S1

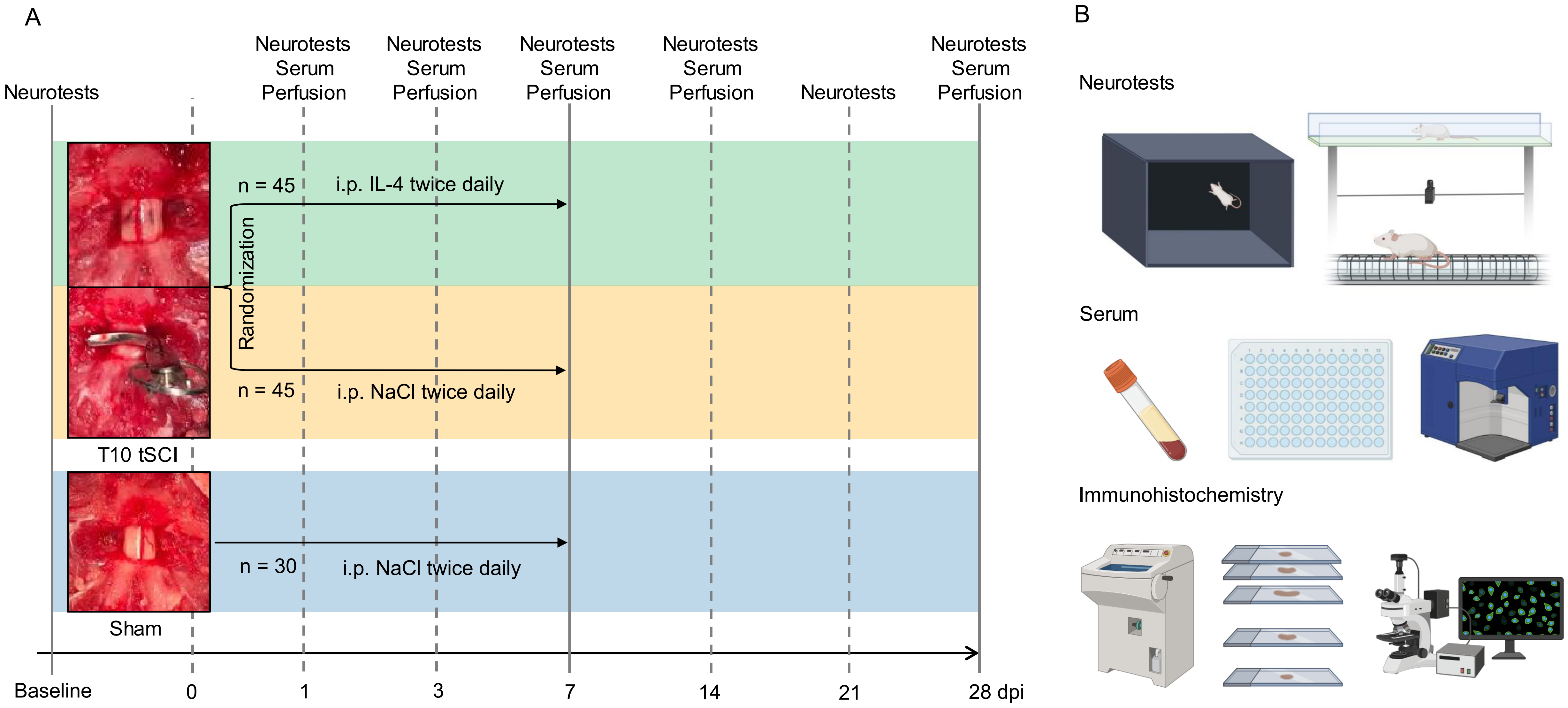

Supplementary Figure S2

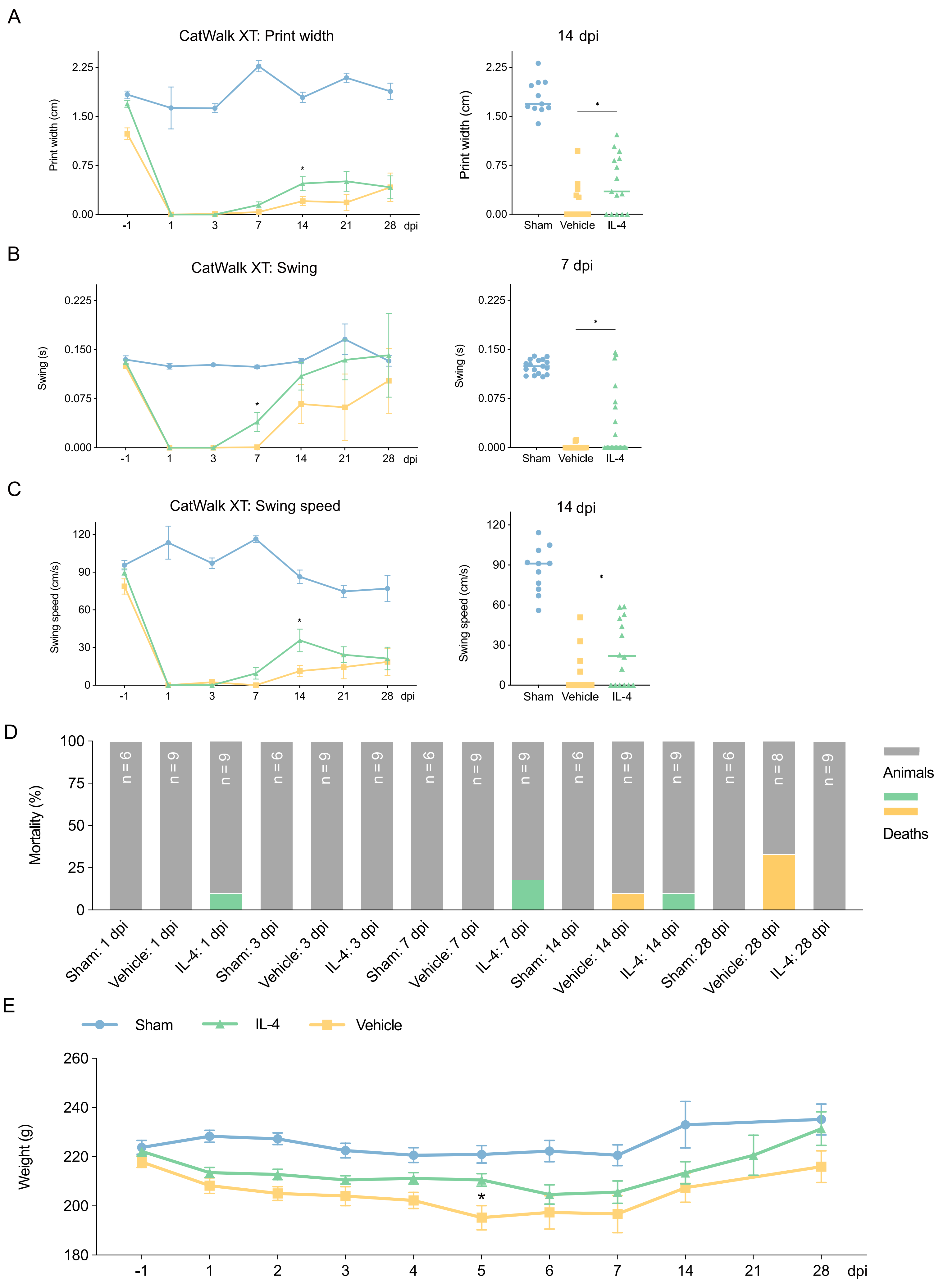

Supplementary Figure S3

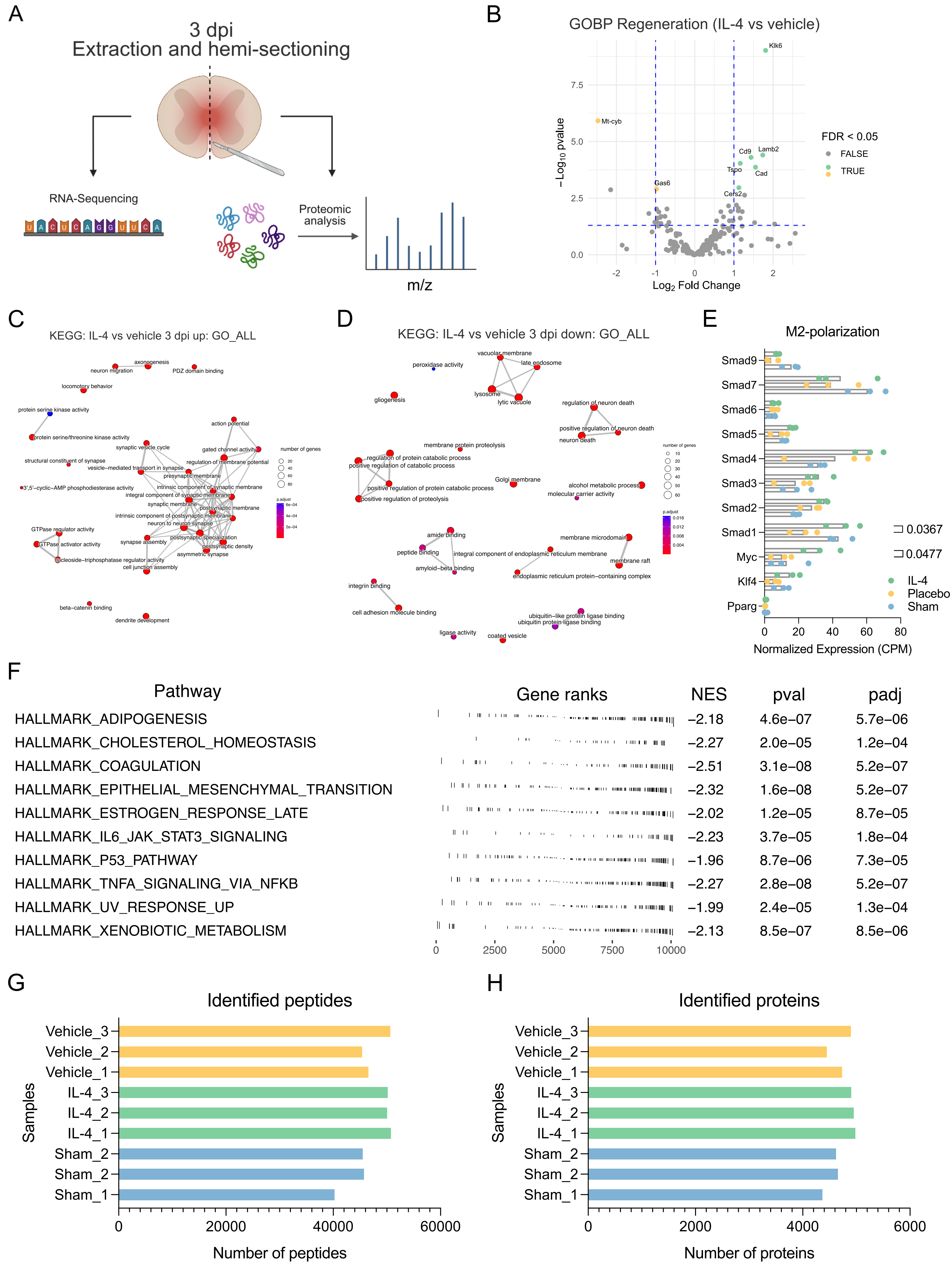

Supplementary Figure S4

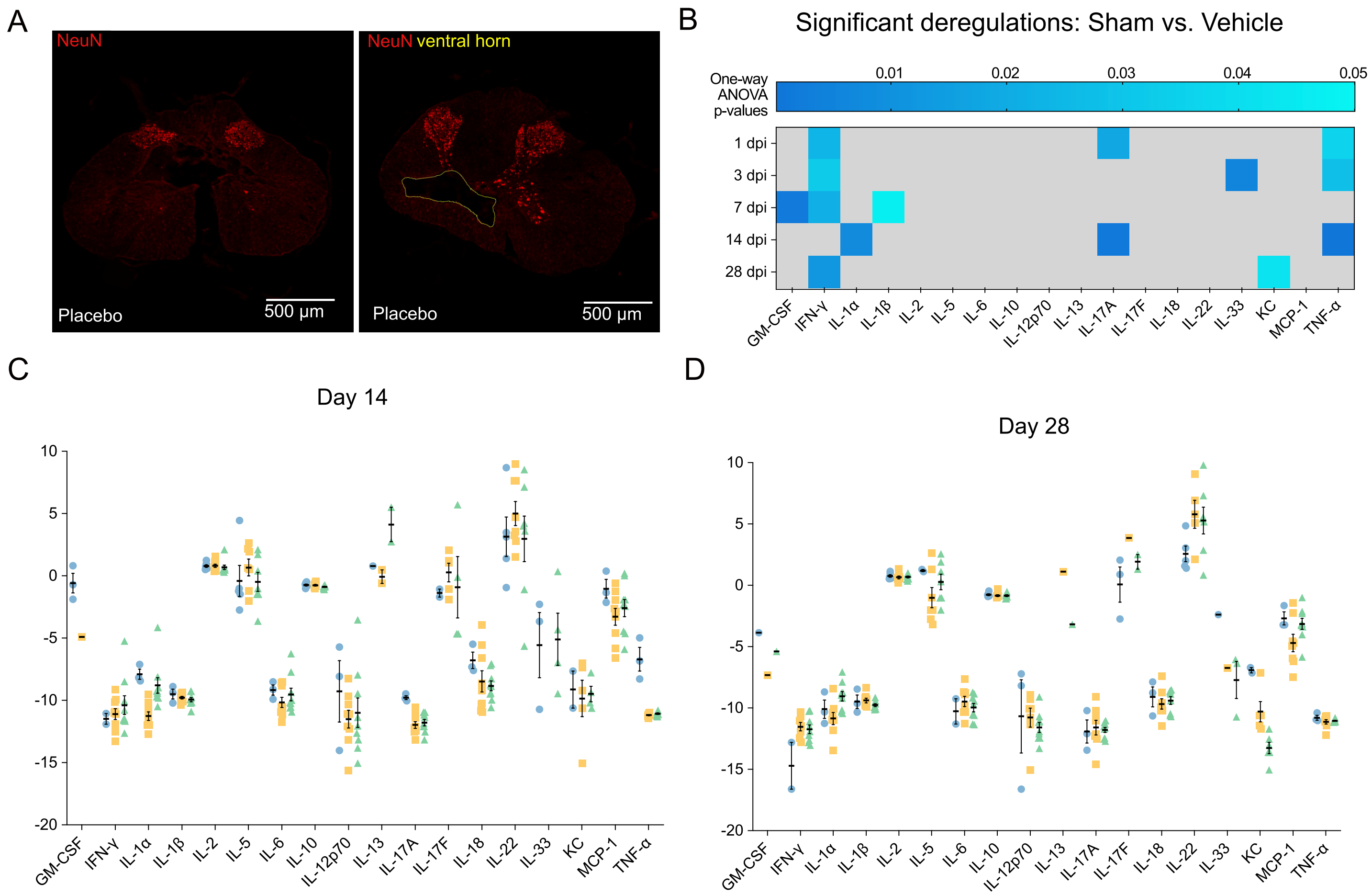

Supplementary Figure S5

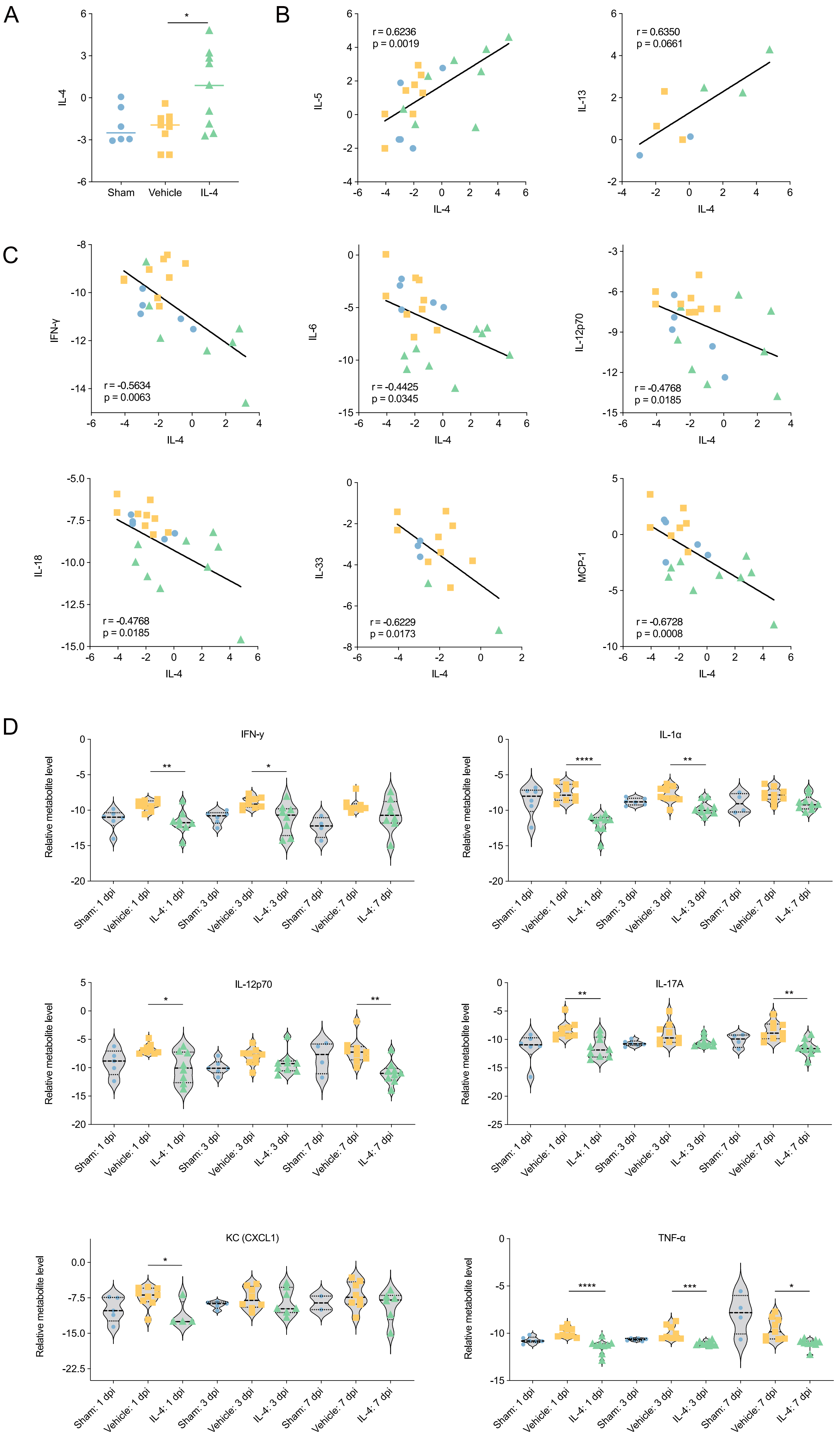

Supplement: Supplementary file 1 — Supplementary figures and table legends. [file thnov16p4726s1.pdf]
